# The function of myostatin in the regulation of fat mass in mammals

**DOI:** 10.1186/s12986-017-0179-1

**Published:** 2017-03-21

**Authors:** Bing Deng, Feng Zhang, Jianghui Wen, Shengqiang Ye, Lixia Wang, Yu Yang, Ping Gong, Siwen Jiang

**Affiliations:** 1 Institute of Animal Husbandry and Veterinary Science, Wuhan Academy of Agricultural Science and Technology, Wuhan, Hubei 430208 People’s Republic of China; 2 Key Laboratory of Swine Genetics and Breeding of the Agricultural Ministry and Key Laboratory of Agricultural Animal Genetics, Breeding and Reproduction of the Ministry of Education, College of Animal Science and Technology, Huazhong Agricultural University, Wuhan, 430070 People’s Republic of China; 3 Wuhan University of Technology, Wuhan, 430074 People’s Republic of China; 4 The Cooperative Innovation Center for Sustainable Pig Production, Wuhan, 430070 China

**Keywords:** Myostatin, Fat, Adipogenesis, Signaling pathway, Regulation

## Abstract

Myostatin (MSTN), also referred to as growth and differentiation factor-8, is a protein secreted in muscle tissues. Researchers believe that its primary function is in negatively regulating muscle because a mutation in its coding region can lead to the famous double muscle trait in cattle. Muscle and adipose tissue develop from the same mesenchymal stem cells, and researchers have found that MSTN is expressed in fat tissues and plays a key role in adipogenesis. Interestingly, MSTN can exert a dual function, either inhibiting or promoting adipogenesis, according to the situation. Due to its potential function in controlling body fat mass, MSTN has attracted the interest of researchers. In this review, we explore its function in regulating adipogenesis in mammals, including preadipocytes, multipotent stem cells and fat mass.

## Background

Adipose tissues, which are mainly composed of adipocytes, play important roles in storage and metabolism [1, 2]. The adipocytes in adipose tissues can be derived from mesenchymal stem cells with the appropriate stimulation. The differentiation process involves two phases: determination, in which multipotent stem cells become adipoblasts, and, differentiation, in which preadipocytes convert to mature adipocytes in the adipogenesis-promoting environment [2]. Myostatin (MSTN), a negative regulator of skeletal muscle growth, can be detected in not only muscle tissues but also adipose tissues [3]. Evidence has now been obtained demonstrating that MSTN could regulate the adipogenesis of mesenchymal stem cells in the determination and differentiation phases [4–6]. In this review, we mainly summarize the structure, tissue distribution, and signal transduction of MSTN and explore the role of MSTN in the adipogenesis of preadipocytes, multipotent stem cells and transgenic animals in mammals.

## The identification of MSTN and its inhibitory effect on muscle differentiation

MSTN, also known as growth and differentiation factor-8, is mainly expressed in skeletal muscle and is a negative regulator of skeletal muscle growth in animals. It was initially identified in 1997 as a member of the transforming growth factor-β superfamily using the degenerate polymerase chain reaction [3]. In the same year, the bovine MSTN gene was mapped to the mh locus by genetic linkage, which strongly suggested that MSTN may be the gene that causes double muscling in cattle [7]. This trait is useful in farm animals because it can dramatically increase muscular mass and improve economic benefits. Further analysis indicated that an 11-bp deletion in the coding region of MSTN in Belgian Blue cattle and a G–A missense mutation in the same region in Piedmontese cattle could cause the double-muscling trait in cattle [8].

Muscle progenitors and myoblast cells can proliferate and terminally differentiate into muscle fibers, which contribute to the growth of muscle mass [9]. The main functions of MSTN in muscle progenitors and myoblasts are self-renewal and differentiation inhibition. For example, in myoblasts, MSTN can inhibit myoblast differentiation into myotubes by preventing myogenic differentiation factor (MyoD) activity and expression via Smad 3 [10, 11]. Similar findings have also been reported in mouse skeletal muscle C2C12 cells, in which MSTN may control muscle mass by inhibiting cell proliferation and DNA and protein synthesis [12]. Further research in satellite cells shows that MSTN could negatively regulate satellite cell activation and control the self-renewal process of satellite cells [13].

## MSTN structure

All transforming growth factor-β (TGF-β) family members contain three distinct domains: an N-terminal signal domain, a propeptide domain and a C-terminal mature peptide [14, 15]. As a member of the TGF-β superfamily, MSTN shares the typical characteristics of other TGF-β superfamily members: 1) a hydrophobic core of amino acids near the N-terminus; 2) a conserved proteolytic processing signal of RSRR in the C-terminus; and 3) cysteine residues in the C-terminal region to facilitate the formation of a “cysteine knot” structure [16–18]. The difference between MSTN and other TGF-β superfamily members is that the nucleotide sequence of the C-terminus is shorter than those in other members [3].

## MSTN tissue expression

MSTN can not only be detected in muscle but can also exert its function in other tissues. Initial research studies in 1997 found that MSTN is predominantly expressed in the muscle tissues of mice and cows [3, 8], but it is also detected in the adipose tissue [3]. Further research showed that MSTN could be detected in mammary glands [19], Purkinje fibers and cardiomyocytes in heart tissue [20], spleen, lymphocytes [21], placenta [22], and even in human uterine gland muscle tissue [23]. Those results indicated that MSTN may exert its function not only in muscle but also in other tissues.

## The function of MSTN in fat formation in mammals

The results of many past studies have indicated that MSTN plays key roles in not only myogenesis but also adipogenesis. The function of MSTN in adipogenesis is controversial. In preadipocytes, MSTN mainly inhibits adipogenesis, whereas it can promote the adipogenesis of pluripotent stem cells. *MSTN* deletion and inhibition in animals mainly lead to increased muscle mass and reduced fat mass. Specific inhibition of *MSTN* in muscle but not adipose tissue inhibits fat mass. Specific overexpression of *MSTN* in adipose tissue increases the metabolic rate and resistance to diet-induced obesity. In the following section, we will explore the role of MSTN in fat formation in mammals.

## Differentiation is inhibited by MSTN in preadipocytes

In different species of preadipocytes, MSTN mainly inhibits cell differentiation. For example, in 3T3-L1 preadipocytes treated with MSTN during differentiation, adipogenesis was significantly inhibited through the regulation of CCAAT/enhancer binding protein (C/EBP) β and peroxisome proliferator-activated receptor γ (PPARγ) [24]. In addition, another adipogenesis transcription factor, *C/EBPɑ*, and lipid metabolism-related genes such as glycerol-3-phosphate dehydrogenase (*GPDH*), diacylglycerol O-acyltransferase (*DGAT*), acyl-CoA synthetase long-chain family member1 (*ACS1*), adipose triglyceride lipase (*ATGL*), and hormone-sensitive lipase (*HSL*) are inhibited by MSTN in 3T3-L1 preadipocyte adipose differentiation [25]. Moreover, the adipogenesis of primary preadipocytes isolated from bovine, porcine fat tissue and intramuscular preadipocytes isolated from porcine longissimus dorsi muscles is also inhibited by treatment with adipogenesis medium plus MSTN [6, 26, 27].

### The inhibition of brown adipogenesis by MSTN

White and brown adipocytes are two types of distinct adipocytes in mammals. White adipocytes mainly store excess energy in large lipid droplet, whereas brown adipocytes contain numerous smaller droplets and burn energy by non-shivering thermogenesis [2, 28]. Research initially focused on the inhibition of adipogenesis by MSTN in white adipocytes. More recent research has revealed that MSTN can also inhibit the differentiation of brown preadipocytes. This process involves TGF-β/Smad3 signaling [29] and Smad3-mediated β-catenin stabilization [30].

Mouse embryonic fibroblasts can differentiate into brown adipose-like cells under specialized adipogenic conditions. Primary mouse embryonic fibroblasts isolated from MSTN-deficient mice exhibit brown adipose-like differentiation and increased lipid metabolism and energy expenditure under specialized brown adipogenic conditions [31]. MSTN treatment in differentiating MSTN-deficient MEF can inhibit key BAT markers (Uncoupling Protein 1 (Ucp1) and PR domain containing 16 (Prdm16)) expression [32, 33]. These results indicate that MSTN can influence brown adipogenesis in mouse embryonic fibroblasts.

## The adipogenesis of pluripotent stem cells is promoted by MSTN

C3H10T(1/2) cells, a mesenchymal fibroblast-like cell line of embryonic origin, have the capacity to undergo differentiation into multiple cell lineages, such as myoblasts, chondrocytes, and adipocytes, after being incubated in different media in vitro [4, 34, 35]. The potential for myogenic differentiation could be inhibited by MSTN, and MSTN can promote the commitment and differentiation of mesenchymal cells into the adipogenic lineages [34].

The function of MSTN in C3H10T(1/2) cell adipogenesis appears to involve driving the cells into a particular state. When cells are induced into adipocytes, DIM, which includes dexamethasone, insulin and isobutyl-1-methylxanthine (IBMX), is always used to trigger adipogenesis [4]. The key component of adipogenesis-inducing medium, dexamethasone, could induce MSTN expression [4, 36]. Pantoja et al. [37] demonstrated that treating C3H10T(1/2) cells with dexamethasone for 48 h followed by IBMX treatment for 48 h was sufficient for adipogenesis, significant differentiation did not occur when C3H10T(1/2) cells were treated with IBMX followed by dexamethasone. Moreover, recombinant MSTN could substitute dexamethasone in the DIM mixture to induce significant levels of adipogenesis in C3H10T(1/2) cells, but not in 3T3-L1 cells (a preadipocyte cell line) [4]. Together, these data show that MSTN may induce adipogenesis in very-early-stage mesenchymal stem cells [4]. This special early stage needs to be clearly confirmed in further studies.

## *MSTN* deletion mainly leads to increased muscle mass, reduced fat mass and resistance to diet-induced obesity

Muscle and adipose tissue develop from the same mesenchymal stem cells [2]. *MSTN* gene function seems to control the switch between adipogenesis and myogenesis. In a mouse model, Lin et al. [38] showed that *MSTN* knockout (KO) led to reduced adipogenesis and consequently decreased leptin secretion, which is associated with increased muscle development. Guo et al. [39] also showed that *MSTN* KO mice exhibited a dramatic increase in muscle mass and reduced fat mass but no changes in the whole-body lipid oxidation rate. By contrast, glucose utilization and insulin sensitivity increased in *MSTN* KO mice. In aging mice, the body fat percentage was also lower in *MSTN* KO compared with WT [40]. Decreased fat accumulation and increased muscle mass were also observed in *MSTN* KO rats [41] and pigs compared with wild type animals [42]. Previous research indicated that adipocytes and myocytes are both derived from the same mesodermal precursor [2]. The diminished fat mass and enhanced muscle in *MSTN* KO mice may be due to rapid depletion of the pool of stem, transit amplifying and progenitor (STP) cells in white adipose tissue (WAT) and brown adipose tissue (BAT) [43].

In addition, the *MSTN* KO mice also exhibited resistance to diet-induced obesity [39]. This resistance phenotype may be due to the transformation of white adipocytes to brown adipocytes. Zhang et al. [44] demonstrated that *MSTN* KO mice are resistant to high-fat diet-induced obesity via an increase in fatty acid oxidation in peripheral tissues and enhanced brown adipose formation in white adipose tissue. Further research indicated that *MSTN* KO mice can drive white adipose tissue into brown adipose tissue with the expression of BAT signature genes, including Ucp1 and peroxisomal proliferator-activated receptor coactivator 1 (Pgc1), and the beige adipocyte markers transmembrane protein 26 (Tmem26) and tumor necrosis factor receptor superfamily member 9 (TNFRSF9, CD137) by activating the AMPK-PGC1-Fndc5 pathway in muscle [32]. miR-34a is also involved in regulating fibronectin type III domain-containing protein (Fndc5) expression in active browning of white adipocytes [45].

## MSTN inhibition also leads to decreased fat tissue in mammals

MSTN inhibition in animals has been investigated and could lead to decreased amounts of fat tissue. When MSTN was suppressed by a propeptide cDNA sequence in transgenic mice, the fat masses in the subcutaneous, epididymal and retroperitoneal areas were significantly less than in WT mice [46]. Similarly, visceral fat was decreased in adult mice upon knockdown of MSTN by siRNA [47]. McPherron et al. [48] found that MSTN inhibition may be more efficacious in reducing adipose weight gain rather than in causing weight loss when MSTN is inhibited by treatment with a soluble MSTN receptor derived from the activin receptor type IIB extracellular domain in high-fat diet-induced mice. Furthermore, the diet-induced obese rats showed reduced body and fat weight using a prepared polyclonal antibody for MSTN [49] and the myostatin antagonist sActRIIB [44]. Dong et al. [50] demonstrated that white adipose tissue is converted to brown adipose tissue and fatty acid oxidation and energy expenditure are promoted when myostatin is inhibited by an anti-myostatin peptibody in HFD-fed mice. This related mechanism is due to muscle-to-fat cross talk by Fndc5 (irisin).

## Tissue-specific MSTN inhibition and over-expression in mice

The inhibition of myostatin signaling in adipose tissue had no effect on body composition in mice fed a standard diet or high-fat diet. By contrast, the inhibition of MSTN signaling in skeletal muscle increased lean mass and decreased fat mass on standard and high-fat diets, as well as resistance to diet-induced obesity [39]. The results indicated that specific inhibition of MSTN in skeletal muscle but not fat tissues can increase resistance to diet-induced obesity.

Adipose tissue-specific MSTN overexpression also increases resistance to diet-induced obesity. Feldman et al. [4] generated aP2-MSTN transgenic mice that express MSTN in fat tissue under the control of the aP2 promoter. The aP2-MSTN transgenic mice exhibited an increased metabolic rate and were resistant to diet-induced obesity. In addition, adipocytes induced by MSTN in both C3h10T1/2 cells and transgenic mice were small and expressed markers characteristic of immature adipocytes.

## MSTN signal transduction in adipogenesis

MSTN, a secreted protein, needs to transmit its signaling into the nucleus via a series cascade reaction to exert its function. Rebbapragada et al. [51] found that MSTN could first bind the type II Ser/Thr kinase receptor (ActRIIB) and then partner with a type I receptor, either activin receptor-like kinase 4 (ALK4 or ActRIB) or ALK5 (TβRI), to induce Smad2/3 phosphorylation to inhibit the adipogenesis of C3H10T1/2 cells. Further research indicated that MSTN can activate Smad3 and the cross-communication of the TGF-β/Smad signal to the Wnt/β-catenin/TCF4 pathway to down-regulate PPARγ, leading to the inhibition of adipogenesis in human bone marrow-derived mesenchymal stem cells and preadipocytes [52]. Recent research indicated that MSTN could regulate MyoD expression to influence PPARγ to exert its function in adipogenesis [53, 54].

## The regulation of *MSTN* gene expression

As an important regulatory element in 5′ upstream regions of genes, the promoter can be bound by transcription factors to regulate gene expression. The analysis of the *MSTN* promoter shows that it can be regulated by many transcriptional factors. Li indicated that MSTN promoter activity is regulated by myocyte enhancer factor 2 in pigs [55]. In cattle and sheep, *MSTN* is regulated by the muscle-related transcription factor myogenic factor 5 (Myf5), myocyte enhancer factor-2 (MEF2) and MyoD [18, 56]. Deng et al. [27] showed that porcine *MSTN* could be upregulated by IBMX, MyoD and PPAR*γ* but down-regulated by C/EBPα and C/EBPβ by analyzing the promoter of the porcine *MSTN* gene. In addition, *MSTN* could be induced by dexamethasone [4, 27]. Those studies indicated that MSTN can be regulated not only by myogenesis-related factors but also by adipogenesis factors.

## Conclusion

As a factor involved in muscle and fat regulation, the function of MSTN in muscle has been widely investigated. In addition, its role in regulating fat mass has also attracted researchers’ interest. It is clear that MSTN has positive and negative effects on adipogenesis depending on the situation. In preadipocytes, MSTN mainly inhibits adipogenesis [23, 26], while it promotes adipogenesis in pluripotent stem cells [4, 37]. Further studies utilizing MSTN transgenic animal models indicated that it mainly promotes fat mass accumulation. For example, *MSTN* gene knockout in animals mainly leads to reduced fat mass and resistance to diet-induced obesity [39, 41]. MSTN inhibition in animals leads to reductions in fat tissues [46, 48, 49]. However, myostatin inhibition in muscle but not adipose tissue inhibits fat mass and improves insulin sensitivity [39]. Adipose tissue-specific MSTN overexpression increases the metabolic rate and resistance to diet-induced obesity [4].

Although many reports show that MSTN participates in the regulation of adipogenesis, more details remain to be elucidated: 1) How does MSTN play different roles in preadipocytes and pluripotent stem cells? 2) To which cellular state does MSTN drive cells? 3) How does MSTN cross-communicate with other adipogenesis-related signaling factors, such as the Wnt signaling pathway?

A better understanding of MSTN in adipogenes is likely to be a novel and promising area for better clinical applications for controlling body and fat weight and animal production.

## Abbreviations

*ACS 1*: acyl-CoA synthetase long-chain family member 1
ActRIIB: Type II Ser/Thr kinase receptor
ALK4: Activin receptor-like kinase 4
*ATGL*: Adipose triglyceride lipase
BAT: Brown adipose tissue
C/EBP: CCAAT/enhancer binding protein
*DGAT*: Diacylglycerol O-acyltransferase
Fndc5: Fibronectin type III domain-containing protein
*GPDH*: Glycerol-3-phosphate dehydrogenase
*HSL*: Hormone-sensitive lipase
KO: Knockout
MEF2: Myocyte enhancer factor-2
MSTN: Myostatin
Myf5: Myogenic factor 5
MyoD: Myogenic differentiation factor
Pgc1: Peroxisomal proliferator-activated receptor coactivator 1
PPARγ: Peroxisome proliferator-activated receptor γ
Prdm16: PR domain containing 16
TGF-β: Transforming growth factor-β
Tmem26: Transmembrane protein 26
TNFRSF9, CD137: Tumor necrosis factor receptor superfamily member 9
Ucp1: Uncoupling Protein 1
WAT: White adipose tissue
WT: Wild-type
